# Serum concentration of antigen-specific IgG can substantially bias interpretation of antibody-dependent phagocytosis assay readout

**DOI:** 10.1016/j.isci.2023.107527

**Published:** 2023-08-03

**Authors:** Russell St. Germain, Emily L. Bossard, Lawrence Corey, Anton M. Sholukh

**Affiliations:** 1 Vaccine and Infectious Diseases Division, Fred Hutch Cancer Research Center, Seattle, WA 98109, USA; 2 Division of Allergy and Infectious Diseases, Department of Medicine, University of Washington, Seattle, WA 98195, USA; 3 Department of Laboratory Medicine and Pathology, University of Washington, Seattle, WA 98195, USA

**Keywords:** Health sciences, Immunology, immune response

## Abstract

Because virus neutralization cannot solely explain vaccine-induced, antibody-mediated protection, antibody effector functions are being considered as a potential correlate of protection (CoP). However, measuring effector functions at a fixed serum dilution for high throughput purposes makes it difficult to distinguish between the effect of serum antibody concentration and antibody properties such as epitopes, subclass, and glycosylation. To address this issue, we evaluated antibody-dependent cellular phagocytosis (ADCP) assay against SARS-CoV-2 spike. Adjustment of serum samples to the same concentration of antigen-specific IgG prior to the ADCP assay revealed concentration-independent differences in ADCP after mRNA vaccination in subjects with and without prior SARS-CoV-2 infection not detectable in assay performed with fixed serum dilution. Phagocytosis measured at different concentrations of spike-specific IgG strongly correlated with the area under the curve (AUC) indicating that ADCP assay can be performed at a standardized antibody concentration for the high throughput necessary for vaccine trial analyses.

## Highlights

• High levels of spike-specific IgG can mask other factors influencing ADCP
• Equalizing spike IgG levels between samples exposes these differences
• mRNA vaccine changes spike IgG glycosylation and ADCP in seropositives
• Equalizing IgG concentrations prior to ADCP assay is recommended

## Graphical abstract

**Figure:**
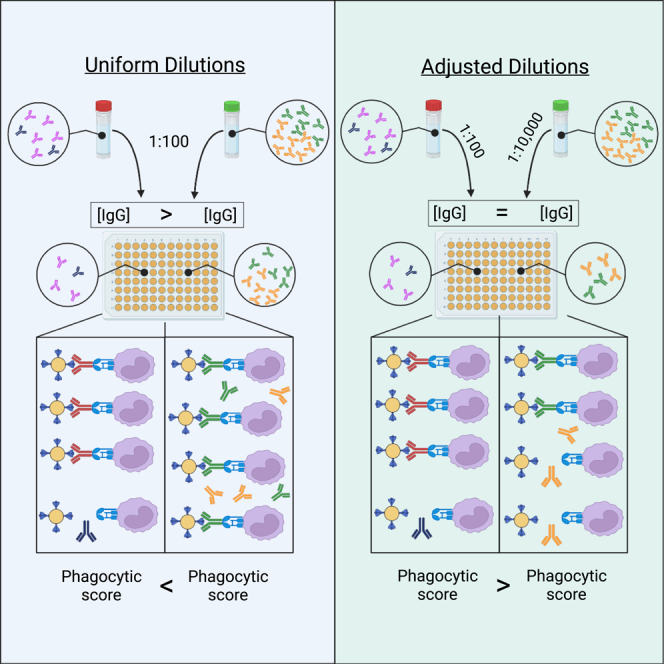

## Introduction

The rapidly unfolding pandemic caused by the severe acute respiratory syndrome coronavirus 2 (SARS-CoV-2) presented unprecedented challenges for vaccine development and testing.^1^^,^^2^ With unparalleled speed, several platforms have been introduced yielding highly successful and efficacious vaccines.^3^ Similarly to the majority of existing vaccines,^4^ protection generated by SARS-CoV-2 vaccines was linked to neutralizing antibodies.^3^^,^^5^^,^^6^ However, neutralization failed to solely explain efficacy of vaccine-induced antibody responses in SARS-CoV-2.^7^ T cell responses and antibody effector functions have been actively investigated as additional correlates of protection (CoPs).^8^^,^^9^

In earlier studies, antibody effector functions such as antibody-dependent cellular phagocytosis (ADCP) and antibody-dependent cellular cytotoxicity (ADCC) have been implicated in protection against multiple viral infections including HIV, Ebola, influenza, Epstein-Barr virus (EBV) and dengue, both in vaccine trials and animal model studies.^10^^,^^11^^,^^12^^,^^13^^,^^14^^,^^15^ High ADCP activity positively correlated with protective vaccine response in human cytomegalovirus (HCMV) vaccine trials.^16^ In COVID-19, increased antibody effector functions were associated with lower disease severity and implicated in protection.^17^^,^^18^ SARS-CoV-2 mRNA vaccines from Moderna and Pfizer/BioNTech elicited antibodies with robust effector functions^9^^,^^19^ and efficacy of subunit SARS-CoV-2 vaccine from NovaVax was found to be dependent on combination of neutralization and effector functions.^20^ Therefore, analysis of antibody effector functions is becoming an essential component of not only pre-clinical vaccine evaluations, but also human vaccine trials and post-hoc CoP analyses.

In contrast to neutralization that is defined by antibody epitope, effector functions depend on both Fab and Fc parts of IgG molecule. The Fab fragment defines magnitude of antibody-mediated cellular responses via abundance and availability of epitopes and affinity of binding, and Fc part determines response type via antibody isotype or subclass and influences response magnitude through glycosylation pattern.^15^^,^^21^^,^^22^^,^^23^ Therefore, the outcome of effector function assay is the product of Fab specificity, Fc properties and concentration of the constituent antibody. The difference in antibody concentrations between compared samples can play a crucial role in assay outcome masking other differences between samples that can be the true cause of functional outcome. This is particularly important for studies involving mRNA vaccines as it has been demonstrated that concentration of antigen-specific antibody after mRNA vaccination can be orders of magnitude higher compared to natural infection or other types of vaccines.^24^^,^^25^^,^^26^

In this study, we compared SARS-CoV-2 ADCP assay performed at the fixed serum dilution versus equalizing serum samples by the concentration of antigen-specific antibody prior to the assay. We demonstrate that high concentration of spike-specific IgG that is observed after vaccination with mRNA vaccines can mask differences in ADCP activity between samples if they are tested at the fixed dilution. Adjustment to the same concentration of spike-specific IgG prior to ADCP assay reveals concentration-independent differences in antibody-mediated phagocytosis.

## Results

### Concentration of spike IgG defines ADCP assay readout

**Figure 1:**
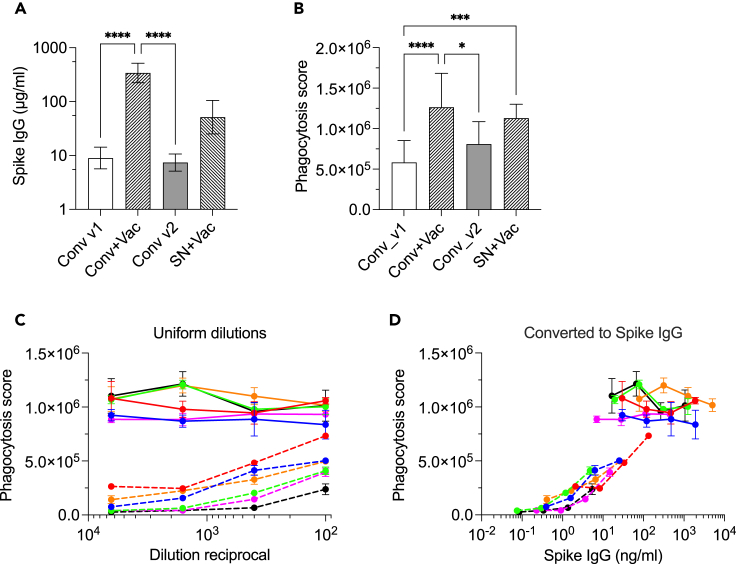
Concentration of spike IgG defines ADCP assay readout (A) Concentration of spike-specific IgG measured using multiplex binding antibody assay (MBAA). Data showed lognormal distribution and statistical analysis was performed with Kruskal-Wallis test with Dunn’s correction for multiple comparisons. (B) ADCP measured at fixed 1:100 plasma dilution. Conv_v1, 25 samples collected at 2 month post-COVID-19; Conv_v2, 9 samples collected at 9 month post-COVID; Conv+Vac, 19 samples collected after one dose of mRNA vaccine in previously infected participants; SN + Vac, 6 samples collected after 2 doses of mRNA vaccine, seronegative individuals. Data shown are mean value ±SD. Because data showed normal distribution, *p* values were determined with Brown-Forsythe and Welch ANOVA test with Dunnett’s T3 multiple comparisons test. (C) Matching plasma samples from 6 participants (depicted by same colors) from Conv_v1 (dashed lines) and Conv+Vac (solid lines) time points were serially titrated 4-fold starting at 1:100. (D) Phagocytosis score obtained in c, was plotted against actual concentration of spike-specific IgG in a sample. ∗p < 0.05; ∗∗p < 0.01; ∗∗∗p < 0.001; ∗∗∗∗p < 0.0001.

ADCP activity of immune serum/plasma is usually measured at the fixed sample dilution, e.g., 1:100, to maintain high throughput of testing.^9^^,^^18^^,^^20^^,^^27^^,^^28^^,^^29^^,^^30^ However, concentration of spike-specific IgG in serum can differ up to 1000-fold making comparison of such sample difficult.^24^^,^^25^^,^^26^ Therefore, we set to test if the outcome of the ADCP assay that was performed at the same, fixed sample dilution is affected by concentration of spike IgG in the sample. We used plasma samples (Table 1) that were collected at the following time points: at about 2 months after COVID-19 infection (indicated as Conv_v1); at 9 months post-COVID-19 (Conv_v2); from individuals that were infected with SARS-CoV-2 and later received one dose of mRNA vaccine (Conv+Vac); from previously noninfected participants that received 2 doses of mRNA vaccine (SN + Vac). Median concentration of spike-specific IgG measured by in-house multiplex binding antibody assay (MBAA) (Figure 1A; Table 1) was the highest among previously infected recipients of mRNA vaccine (Conv+Vac), 449.6 μg/mL (95%CI [189.9; 586.4]). During early (Conv_v1) and late (Conv_v2) convalescence, concentration of spike IgG was about 45-fold lower and there was a slight decline between 2- and 9-month post-infection from 9.9 to 7.6 μg/mL. Seronegative vaccinees had median concentration of 46.8 μg/mL (95%CI [25.2; 174.8]). Thus, difference in concentration of antigen-specific IgG between samples was between about 4- to 45-fold.

**Table 1:** Group demographics and concentration of spike IgG

Group	No. Individuals	Status	Median age, years	Female, %	Male, %	Sample overlap with Conv_v1	Median spike-specific IgG (μg/mL)	95% CI
Conv_v1	25	Convalescent	53.9	40	60	N/A	9.92	5.54
		2 months						21.31
Conv_v2	9	Convalescent	52	56	44	6	7.59	4.88
		9 months						11.48
Conv+Vac	19	Convalescent	58	32	68	19	449.60	189.90
		9 months + Vaccinated						586.40
SN + Vac	6	Seronegative + Vaccinated	43.5	50	50	N/A	46.80	25.21
								174.80

Next, we measured antibody-mediated phagocytosis scores at 1:100 plasma dilution for all samples (Figure 1B). In previously infected vaccine recipients (Conv+Vac), ADCP was significantly higher than in convalescent individuals (Conv_v1 and Conv_v2) but did not significantly differ from seronegative vaccinees (SN + Vac), similar to earlier observations.^9^ In late convalescence (Conv_v2) despite decline of spike IgG, ADCP activity was higher compared to early convalescence, although not statistically significant. In seronegative vaccinees (SN + Vac) ADCP was comparable to that in Conv+Vac despite 10-fold lower concentration of spike-specific IgG. Notably, the difference in spike IgG between Conv_v1 and Conv+Vac was about 45.3-fold while phagocytic score differed only about 2-fold. This can indicate that the ADCP assay reached the upper limit of quantification and therefore cannot properly reflect differences between tested samples. Therefore, we measured ADCP activity in paired samples collected at 2 months post-infection (Conv_v1) and after one dose of mRNA vaccine (Conv+Vac) using 4-fold serial plasma titration with starting dilution of 1:100 (Figure 1C). While dose-dependent titration of phagocytosis score was observed in Conv_v1 samples (dashed lines), in Conv+Vac no titration was seen (solid lines). Following plotting of phagocytosis score over actual concentrations of spike IgG calculated from dilution reciprocals further illustrates that measurement for Conv+Vac were taken at the upper plateau of the titration curve (Figure 1D). Notably, for three samples (red, blue, and magenta), titration curves overlapped over a narrow range of IgG concentrations allowing direct comparison of ADCP activity taken at the approximately same concentration of antigen-specific IgG. Here, phagocytosis score differed drastically between Conv_v1 and Conv+Vac samples. Because IgG concentration is almost identical for the compared samples the difference in ADCP can be attributed to the differences in antibody repertoire, specificity and/or glycosylation as a result of vaccination.

### Equalizing spike IgG concentrations reveals concentration-independent differences in ADCP activity

**Figure 2:**
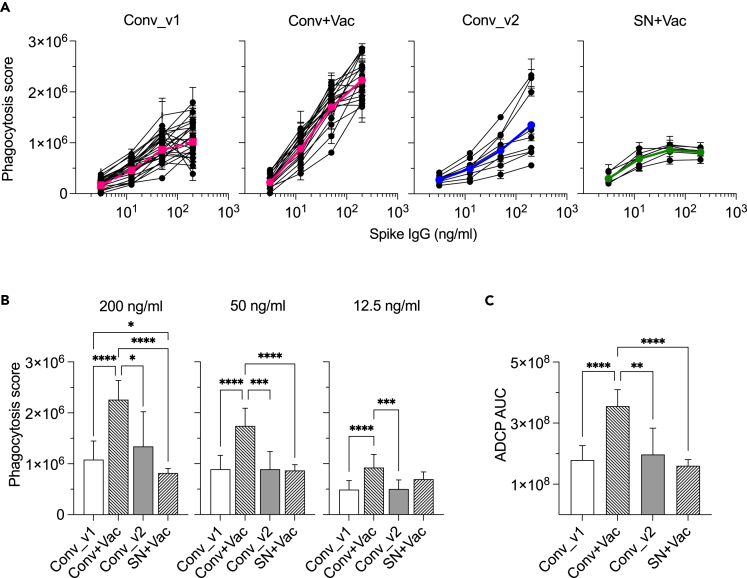
Adjustment of samples to the same concentration of spike-specific IgG reveals concentration-independent differences in ADCP activity All samples were brought to 200 ng/mL of spike-specific IgG and titrated 4-fold. (A) Geometric mean curves depicted in colors and individual samples are overlaid in black. Conv_v1, samples collected at 2-month post-COVID-19 (n = 25); Conv+Vac, samples collected after one dose of mRNA vaccine in previously infected participants (n = 19); Conv_v2, samples collected at 9-month post-COVID (n = 9); SN + Vac, samples collected after 2 doses of mRNA vaccine in seronegative subjects (n = 6). (B) Brown-Forsythe and Welch ANOVA test with Dunnett’s T3 multiple comparisons test comparing phagocytic score at an indicated concentration of spike IgG. (C) Comparison of AUC for curves on panel (A). Data shown are mean value ±SD. *p* values were determined with Brown-Forsythe and Welch ANOVA test with Dunnett’s T3 multiple comparisons test. ∗p < 0.05; ∗∗p < 0.01; ∗∗∗p < 0.001; ∗∗∗∗p < 0.0001.

Observation of different ADCP activity at the similar concentration of spike-specific IgG prompted us to compare plasma samples adjusted to 200 ng/mL of spike-specific IgG. ADCP was measured in 4-fold serial dilutions (Figure 2). In contrast to the previous experiment (Figure 1C), all samples yielded dose dependent ADCP curves (Figure 2A). ADCP activity in previously infected recipients of mRNA vaccine (Conv+Vac) grew at the highest rate compared to other groups. Notably, ADCP activity in samples collected from seronegative mRNA vaccine recipients (SN + Vac) plateaued early, after 50 ng/mL of spike IgG. Mean phagocytosis score in Conv+Vac was significantly higher than in other groups for all tested concentrations of spike IgG (Figure 2B). There was also significantly higher ADCP in early convalescent compared to seronegative vaccinees although this was found only at 200 ng/mL of spike IgG. Comparison of area under the curve (AUC) for geometric mean ADCP titration curves between groups (Figure 1C) corroborated findings with phagocytosis score.

Results of the experiment with sample adjustment to the same concentration of spike IgG contradict outcomes of fixed plasma dilution approach (Figure 1B) that 1) showed similar ADCP in SN + Vac and Conv+Vac samples despite 10-fold difference in spike IgG concentration and 2) demonstrated significantly higher ADCP activity in SN + Vac compared to Conv_v1. Therefore, adjustment of antigen-specific IgG to the same concentration between samples in ADCP assay can reveal qualitative changes in antibody response over time or because of vaccination that could not be detected when serum is tested at the fixed dilution.

### Phagocytosis score at the fixed IgG concentration correlates with area under the curve

**Figure 3:**
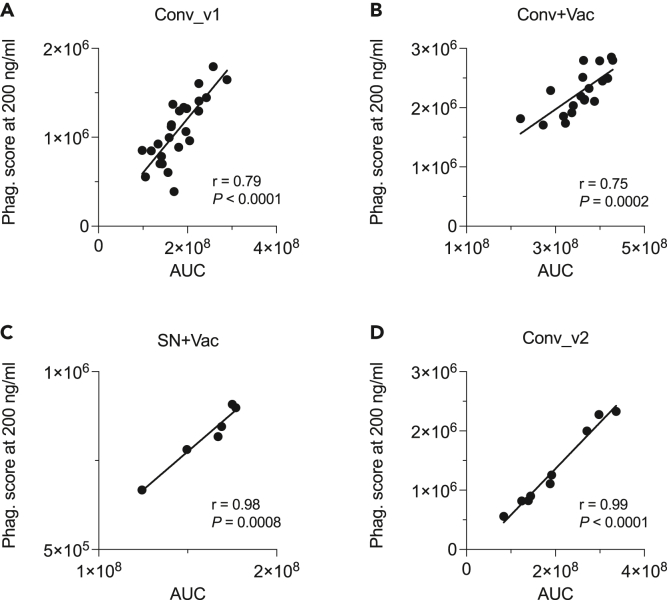
Deming linear regression and Pearson correlation for AUC and phagocytosis score at 200 ng/mL of spike-specific IgG, related to Figures S1 and S2 (A) Samples collected at 2-month post-COVID-19 (n = 25). (B) Samples collected after one dose of mRNA vaccine in previously infected participants (n = 19). (C) Samples collected after 2 doses of mRNA vaccine in seronegative subjects (n = 6). (D) Samples collected at 9-month post-COVIDS (n = 9). Values for Pearson’s r and two-tailed *P* are depicted on graph inserts.

Because titration required to obtain AUC is less preferable for high throughput analyses, we tested whether measurement of ADCP activity at the fixed concentration of antigen-specific IgG can be used instead. Pearson correlation revealed strong positive association between AUC and phagocytosis score at 200 ng/mL of spike-specific IgG for all groups (Figure 3). Similar association was also observed between AUC and phagocytosis score at 50 ng/mL and 12.5 ng/mL concentrations of spike IgG, respectively (Figure S1. Deming linear regression and Pearson correlation for AUC and phagocytosis score at 50 ng/mL of spike-specific IgG; Figure S2. Deming linear regression and Pearson correlation for AUC and phagocytosis score at 12.5 ng/mL of spike-specific IgG, Related to Figure 3), suggesting that comparison between samples can be performed at virtually any concentration of antigen-specific IgG allowing to obtain phagocytosis score readout within assay limits. These data also indicate that measurement of phagocytosis score at the fixed concentration of antigen-specific IgG can be used to substitute AUC approach when assay throughput is needed.

### Deglycosylation differentially changes ADCP activity in vaccinated vs. infected samples

Structure of IgG Fc glycans attenuates IgG binding to Fcγ receptors (FcγRs).^22^ Lower level of fucosylation increases IgG binding to FcγRIII, ADCC-mediating receptors, up to 17-fold and additional hyper-galactosylation increases it further up to 40-fold.^31^ Galactosylation and presence of bisection together with fucosylation were reported to increase IgG binding to the ADCP receptor FcγRIIA at about 2-fold.^31^^,^^32^^,^^33^ From the other hand, highly statistically significant increase in binding of spike-specific antibody to FcγRIIA and FcγRIIIB was reported after the 1st dose of mRNA vaccines in hybrid-immune individuals.^34^ Infected individuals have higher, although not statistically significant, content of GlcNAc bisection especially profound in severe COVID compared to vaccine recipients.^35^ In our hybrid immunity cohort, after mRNA vaccination ADCP activity was significantly higher compared to other groups, therefore we hypothesized that this difference can be due to IgG glycosylation.

To address that, we isolated plasma IgG from six samples from each group and treated it with Endo S, PNGase F and α1-2,4,6 Fucosidase O glycosidases. PNGase F cleaves between the innermost GlcNAc and Asn residues. Endo S specifically removes Asn-linked glycan leaving first GlcNAc attached to Asn residue that can be either free or fucosylated. This fucose can be removed by Fucosidase O, an exoglycosidase that catalyzes the hydrolysis of terminal α1-2, α1-4 and α1-6 linked fucose. Complete deglycosylation of IgG should inhibit its binding to FcγRII and thus reduce ADCP activity^36^^,^^37^ while differential sensitivity of glycans to hydrolysis by Endo S and PNGase F^38^^,^^39^^,^^40^ can reveal differences in glycosylation patterns.^41^^,^^42^

**Figure 4:**
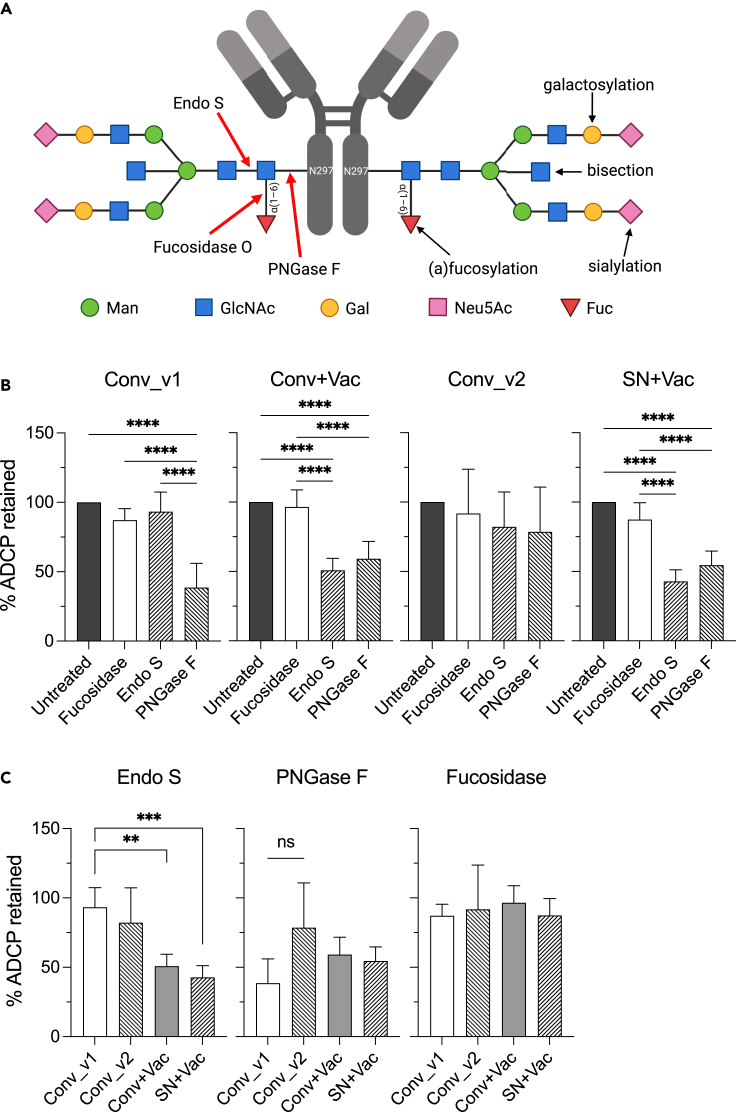
Deglycosylation differentially changes ADCP activity in vaccinated vs infected samples Total IgG was isolated from 6 samples per group, treated with Endo S, PNGase F and α1-2,4,6 Fucosidase O and subjected to ADCP assay. (A) Schematic representation of IgG glycan with possible modifications (right glycan, black arrows) and cleavage sites for exoglycosidases (left glycan, red arrows). Man, mannose; GlcNAc, N-acetylglucosamine; gal, galactose; Neu5Ac, N-acetylneuraminic acid; Fuc, fucose. Created with BioRender.com. (B) Comparison of effects of different glycosylases on ADCP activity within each group by ordinary one-way ANOVA with Turkey’s multiple comparison test. (C) Comparison of ADCP between different groups by Brown-Forsythe and Welch ANOVA test with Dunnett’s T3 multiple comparisons test. y axis represents percent ADCP activity after the treatment with indicated glycosidase compared to the untreated IgG. Data shown are mean value ±SD. ∗p < 0.05; ∗∗p < 0.01; ∗∗∗p < 0.001; ∗∗∗∗p < 0.0001.

Because Fucosidase only removes fucose at the initial GlcNAc, this treatment can affect binding to FcγRIII but not FcγRIIA as the latter does not depend on fucosylation.^31^ Indeed, Fucosidase O treatment did not inhibit ADCP as 100% ADCP activity was retained after the treatment (Figure 4A). PNGase F treatment decreased ADCP activity in Conv_v1, Conv+Vac and SN + Vac groups 2-fold but Endo S treatment affected only vaccine recipients regardless of infection status (Conv+Vac and SN + Vac). Ability of IgG from the late convalescent group (Conv_v2) to mediate ADCP was affected neither by Endo S nor by PNGase F treatment. Comparison of ADCP activity mediated by deglycosylated IgG between groups further revealed that ADCP after Endo S treatment was significantly lower among mRNA vaccinees than in convalescence but the relative resistance of IgG from late convalescent samples to PNGase F treatment was not statistically significant. Therefore, differential decrease in ADCP in response to PNGase F and Endo S treatment indicates on differences in IgG glycan composition between convalescent and vaccinated individuals.

## Discussion

This study provides an evaluation of different approaches to measure, analyze, and compare the capacity of serum IgG to mediate phagocytosis in samples with a broad range of antigen-specific IgG concentrations. We show that concentration of antigen-specific IgG has a much larger impact on ADCP assay readout than properties of IgG molecule that can change over time or as a result of vaccination. We also demonstrate that equilibration of samples by concentration of antigen-specific IgG prior to ADCP assay is needed to adequately evaluate differences in the capacity of serum IgG to mediate phagocytosis between samples.

RBD-specific IgG induced by mRNA vaccines showed higher level of sialylation, fucosylation and lower level of bisection compared to total IgG and to IgG from infected persons.^35^ From the other hand, fucosylated glycans have the fastest deglycosylation rate by PNGase F compared to all other types and glycans with bisection are more resistant to Endo S-mediated hydrolysis.^38^^,^^39^^,^^40^ Taken together, this can explain greater impact of both PNGase F and Endo S on ADCP activity among vaccine recipients as IgG induced by mRNA vaccination is fucosylated and contain less of glycans with bisection. Our data are also in line with recent results by Van Coillie et al. that demonstrated substantial decrease in glycan bisection of spike-specific IgG after the first dose of mRNA vaccine in previously infected individuals.^43^ Discordance between ADCP level in experimental groups (Figure 2) and effect of deglycosylation on ADCP activity (Figure 4) rather suggests that glycosylation pattern is not the cause of the observed difference. Monoclonal antibodies with different epitope specificity to SARS-CoV-2 have been shown to differ in their capacity to mediate phagocytosis at the same tested concentration.^44^ Because concentration of spike IgG was equalized between samples, we can speculate that properties of antibody response such as avidity, affinity, and epitope specificity but not IgG glycosylation pattern define observed increase in ADCP activity after mRNA vaccination in previously infected persons.

Very high concentration of antigen-specific antibody in a sample such as seen after vaccination with mRNA vaccines^24^^,^^45^ can influence ADCP assay readout much more strongly than actual changes to the IgG molecule or epitope specificity. Vaccinated individuals infected with Omicron BA.1 have significantly higher levels of ADCP compared with unvaccinated individuals but the difference exactly mirrors levels of binding antibodies suggesting that it is the main cause of the observed difference.^28^ Another study has found ADCC activity is higher in convalescents compared with seronegative recipients after one dose of mRNA vaccine, but despite that ADCP activity followed the trajectory of binding antibodies, authors concluded that Fc functions in the second group were superior.^46^

To eliminate the impact of antibody concentration on the measured function in neutralization and antigen binding assays, samples are serially diluted to establish either 50% effective dilution or endpoint titer. In contrast, ADCP activity is usually analyzed at fixed serum/plasma dilution e.g., 1:100.^18^^,^^47^ We show that with this approach it is impossible to define whether the difference in ADCP activity between samples is the result of higher antibody concentration, functional changes to IgG molecule, or differences in epitope specificity. Similar to our findings, normalization of ADCP and ADCC assay readouts to the content of spike-specific antibody eliminated the difference between infection-induced and vaccination-induced antibodies in seronegative individuals that was seen when effector functions were measured at fixed sample dilution.^48^

In conclusion, our data provide evidence for the necessity to account for the content of antigen-specific immunoglobulin in a sample prior to not only ADCP assay but likely to all assays testing antibody effector functions. This can be achieved by preceding effector function measurement with binding antibody assay that provides quantitative output such as titer or concentration in any standardized units that should be used for sample equilibration by the content of antigen-specific IgG. In high throughput settings, such adjustment can be handled via specific programming of sample handling robots or by grouping samples with similar concentration of antigen-specific IgG. In regard to mRNA vaccines that induce unseen before high serum levels of antigen-specific antibodies, such modification to effector function assay can be critical for correct evaluation of vaccine-induced immune responses.

### Limitations of the study

The limitation of our study is the relatively small cohort of seronegative vaccine recipients. However, previous studies showed the same relationship in ADCP activity between seronegative vaccine recipients and convalescent individuals indirectly supporting our findings.^28^^,^^48^ Our study is also limited in analysis of the possible role of IgG subclass composition in modulation ADCP activity. However, no significant difference was found between mild infection, vaccination, and hybrid immunity by levels of different IgG subclasses to SARS-CoV-2 antigens suggesting that total IgG can be used to compare effector functions between different scenarios of humoral immunity to SARS-CoV-2.^35^^,^^49^ Sample titration for ADCP curves was limited to four dilutions of spike-specific IgG and thus did not allow for establishing full-range dose-response curve needed to properly impute phagocytosis score per μg of spike IgG, which can be used as a better parameter for characterizing changes in IgG molecule underpinning the changes in ADCP activity.

## STAR★Methods

### Key resources table

**Table:** 

REAGENT or RESOURCE	SOURCE	IDENTIFIER
**Antibodies**		

Anti-human IgG Fc-PE	SouthernBiotech	Cat# 2040–09; RRID: AB_2795648
Human IgG	Sigma-Aldrich	Cat# I2511; RRID: AB_1163604
Anti-human IgG Fab-specific	SouthernBiotech	Cat# 2085–01; RRID: AB_2795785

**Biological samples**		

Vaccinated seropositive	FHCC	IRB#10453
Seattle-area participants	University of Washington	NCT04338360
Pooled anti–SARS-CoV-2 product	University of Washington	NCT04344977
Vaccinated seronegative volunteers	FHCC	IRB#3612

**Chemicals, peptides, and recombinant proteins**		

RPMI 1640	Gibco	11875–093
Sodium pyruvate	Gibco	11360–070
Sodium bicarbonate	Gibco	25080–094
HEPES	Gibco	15630–080
PenStrep	Gibco	15070–063
Fetal bovine serum	HyClone	SH30071.03
PBS	Gibco	14190–144
Tween 20	Sigma-Aldrich	P7949-500ML
Sodium Azide	Millipore Sigma	S8032
One step TMB solution	Thermo Scientific	34028
SARS-CoV-2 spike trimer	BEI Resources	Cat# NR-53524; GenPept: QJE37812
SARS-CoV-2 spike trimer	The Stamatatos Lab, VIDD, FHCC	GenBank: QJE37812.1
SARS-CoV-2 RBD	BEI Resources	Cat# NR-52366; GenPept: QHD43416
OC43 spike	Sino Biological	40607-V08B
HKU1 spike	Sino Biological	40606-V08B
NL63 spike	Sino Biological	40604-V08B
229E spike	Sino Biological	40605-V08B
Tetanus toxoid	Enzo	ALX-630-108
Paraformaldehyde	Boster	AR1068
Sulfo-NHS-LC-biotin	Thermo Scientific	A39257
Sulfo-NHS	Thermo Scientific	A39269
EDC	Thermo Scientific	77149
Endo S	NEB	P0741S
PNGase F	NEB	P0708S
α1-2,4,6 Fucosidase O	NEB	P0749S
SimplyBlue Safe Stain	Invitrogen	LC6060
Bolt LDS sample buffer	Thermo Scientific	B0007

HRP-conjugated streptavidin	Thermo Scientific	N100
**Critical commercial assays**		

Melon gel IgG purification kit	Thermo Scientific	45212

**Experimental models: Cell lines**		

THP-1 monocytes	ATCC	TIB-202

**Software and algorithms**		

GraphPad Prism	GraphPad Software	v.9.4.0
FlowJo	BD Biosciences	V10.7.1

**Other**		

Zeba spin desalting column	Thermo Scientific	89882
96-well plate Immulon 2 HB plate	Thermo Scientific	3455
Blotting-grade blocker	Bio-rad	1706404
NeutrAvidin-labeled microspheres	Invitrogen	F8776
Bovine serum albumin	SAFC Biosciences	B4287-100G
Bio-Plex Pro Magnetic COOH beads	Bio-Rad	MC10027-01
Laser FacsCanto II Flow Cytometer	BD BioSciences	N/A
Bio-Plex 200 System	Bio-Rad	N/A
Bolt 4–12% Bis-Tris Plus protein gels	Invitrogen	NW04122BOX
NanoDrop 1000 Spectrophotometer	Thermo Scientific	N/A

### Resource availability

#### Lead contact

Requests for resources and reagents should be directed to and will be fulfilled by Anton Sholukh (asholukh@fredhutch.org).

#### Materials availability

This study did not generate new unique reagents.

### Experimental model and study participant details

#### Plasma samples

Informed consent was obtained from all human subjects, including blood donors, involved in the study. Plasma samples from SARS-CoV-2 seropositive, vaccinated individuals were obtained from Fred Hutchinson Cancer Center (FHCC) repository assembled from a COVID-19 seroepidemiology study conducted in a single county in the western US, FHCRC IRB#10453^50^^,^^51^ and from University of Washington repository assembled from Seattle-area participants that were recruited for potential donation of single-donor plasma units (ClinicalTrials.gov: NCT04338360), and plasma for manufacture of a pooled anti–SARS-CoV-2 product (NCT04344977).^52^^,^^53^ Plasma from seronegative vaccine recipients was obtained from either the same studies or from healthy volunteers at FHCRC (approved by the FHCRC Institutional Review Board, IRB#3612).

#### Cell culture

THP-1 monocytes were kept in R10 media, RPMI 1640 supplemented with sodium pyruvate, sodium bicarbonate, HEPES, PenStrep, and 10% fetal bovine serum. Cells were incubated at 37°C in the presence of 5% CO_2_ and kept at a concentration below 5 × 10^5^ cells/ml to reduce assay variability.

### Method details

#### Multiplex binding antibody assay (MBAA) for SARS-CoV-2 IgG

MBAA was performed as initially described.^51^ Briefly, SARS-CoV-2 spike protein and RBD along with spike proteins of OC43, HKU1, NL63 and 229E and tetanus toxoid were conjugated to Bio-Plex Pro Magnetic COOH beads activated by incubation with 1-ethyl-3-[3-dimethylaminopropyl]carbodiimide-HCL (EDC) and N-hydroxylsulfosuccinimide (Sulfo-NHS). Post activation, beads were washed 3 times and incubated with 10 μg of protein antigen per 2.5 × 10^6^ beads/mL for 2 h at room temperature. After conjugation the beads were washed three times with wash buffer (PBS, 0.05% Tween 20, 1% BSA, 0.1% NaN_3_) and resuspended to achieve 1 × 10^7^ beads/mL. Bead-protein conjugates were stored protected from light at 4°C. Efficiency of conjugation was assessed by binding with established internal quality controls.

To measure concentration of antigen-specific IgG in plasma samples, two replicate dilution series of plasma were incubated with MagPlex beads conjugated with protein antigens for 1 h at room temperature and after three washes with PBS supplemented with 0.05% Tween 20 beads were incubated for another hour with secondary, anti-human IgG Fc-PE. Background was established by measuring the mean fluorescence intensity (MFI) of beads conjugated to antigens incubated in assay buffer and subtracted from all readings. Pooled sera from normal human donors collected in 2015–2016 was included as the negative control for SARS-CoV-2 antigens. Convalescent plasma from a subject with PCR-confirmed severe COVID-19 was used as a positive control.

To impute concentration of antigen-specific IgG, we measured the amount of IgG that was bound to the antigen beads using a standard curve performed with serial dilutions of standard human IgG. The latter was captured by MagPlex beads conjugated with Fab-specific anti-human IgG and probed with secondary, anti-human IgG Fc-PE, same as for antigen-specific IgG measurements. MFI readings and associated IgG concentrations were fitted to a four-parameter logistic curve (4PL) using the R packages *nCal* and *drc* or by 5PL curve algorithm in Bio-Plex Manager software (Bio-Rad) as both yeilded congruent results. Concentration of antigen-specific IgG was then calculated using the resulting standard curves.

#### Biotinylation

Stabilized trimer of SARS-CoV-2 spike (Wuhan strain)^51^ was biotinylated with 0.1 mM sulfo-NHS-LC-biotin at room temperature for 30 min on rotator. Biotin was removed by desalting on Zeba spin desalting column equilibrated with PBS.

#### Biotinylated protein ELISA

Biotinylated and original spike proteins were adsorbed on 96-well plate Immulon 2 HB plate from 1 μg/mL solution in PBS overnight at 4°C. Next day, plate was washed with wash buffer (PBS, 0.05% Tween 20) and blocked with PBS with 0.05% Tween 20 and 5% Blotting-Grade Blocker for 1 h at room temperature. Washed plate was then incubated with streptavidin-horseradish peroxidase (HRP) for 1 h at room temperature. After the wash, plate was developed with One Step TMB solution, absorbance at 450 nm was collected on Victor X3 plate reader (PerkinElmer). Successful biotinylation was confirmed if signal with biotinylated spike protein was >1 A.U. after background subtraction.

#### Antibody-dependent cellular phagocytosis assay

Assay was adapted from described earlier.^54^ NeutrAvidin-labeled Microspheres were conjugated with biotinylated SARS-CoV-2 spike protein in the ratio of 26 ng spike per 10^6^ beads for 3 h at 4°C. Protein-bound beads were washed in Assay Buffer (PBS, 1% BSA) and brought to a final concentration of 2 × 10^4^ beads/μL.

Plasma samples were serially diluted 4-fold in FBS-free RPMI media with starting dilution of 1:100 for “uniform” dilution approach. For the “equalized” dilution scheme, initial dilution factor was calculated to achieve concentration of spike-specific IgG in plasma samples of 200 ng/mL. Then samples were serially diluted 4-fold. Spike IgG concentration was measured via MBAA.

Spike-conjugated microbeads were mixed with plasma dilutions in 1:1 volume ratio and incubated for 1 h at room temperature. Plates with plasma-beads mixture were centrifuged, supernatant was removed, and beads were resuspended in 100 μL of 10^5^ cells/mL THP-1 cells suspension in R10 media. Plates were incubated at 37°C for 18 h, then centrifuged to remove supernatant and fixed with 4% paraformaldehyde (PFA) for 15 min. PFA was removed by centrifugation and cells were resuspended in Assay Buffer. Uptake of beads by THP-1 cells was measured via flow cytometry on Laser FacsCanto II Flow Cytometer (BD BioSciences), and analysis was performed using FlowJo V10.7.1 (BD BioSciences). MFI value of FITC-positive cells from background wells containing THP-1 cells and spike-conjugated beads was subtracted from experimental samples and phagocytosis score (PS) was calculated using the equation:

PhagocytosisScore=%ofFITCpositivecells×MFIofFITCpositivecells

#### Deglycosylation of purified IgG from serum samples

IgG was purified from 6 serum samples from each group using suspension Melon gel IgG purification kit according to manufacturer protocol. Briefly, serum was diluted in Melon gel purification buffer and incubated with Melon gel for 5 min at room temperature followed by centrifugation. Supernatant containing purified IgG was collected. Buffer exchange to PBS was performed using Zeba spin desalting columns. Pure IgG was confirmed on SDS protein gel. Total IgG concentration was measured at 280 nm on Nanodrop 1000 (ThermoScientific) and spike-specific IgG concentration was measured via MBAA.

Purified IgG was diluted to obtain 1000 ng/mL of spike IgG, then incubated in three separate reactions with Endo S, PNGase F, and α1-2.4.6 Fucosidase O endoglycosidases (all from NEB). Enzyme-IgG mixtures were made according to manufacturer protocols. For Endo S, 20 ng IgG was mixed with 400 units of enzyme and incubated at 37°C for 1 h. For PNGase F, 20 ng IgG was mixed with 1000 units of enzyme and incubated at 37°C for 20 h. For Fucosidase, 10 ng IgG was mixed with 4 units of enzyme and incubated at 37°C for 18 h. After incubations, samples were taken directly into the ADCP assay.

### Quantification and statistical analysis

Statistical analysis was performed in GraphPad Prism (v.9.4.0; GraphPad Software, San Diego, CA). Sample distribution was analyzed using normality and lognormality tests and relative likelihood of sampling from a Gaussian vs. lognormal distribution was computed. Samples with normal distribution were compared using Brown-Forsythe and Welch ANOVA test with Dunnett’s T3 multiple comparisons test (∗p < 0.05; ∗∗p < 0.01; ∗∗∗p < 0.001; ∗∗∗∗p < 0.0001). For Figure 4A ordinary one-way ANOVA with Turkey’s correction for multiple comparisons test was used because comparisons was preformed against “untreated” samples set at 100%. Samples with lognormal distribution were compared via Kruskal-Wallis test with Dunn’s correction for multiple comparisons.

Pearson correlation coefficients and two-tailed *p* values were determined for the relationship between ADCP AUC and phagocytosis score at a given ng/ml of spike-specific IgG, according to data distributions and expectation of a monotonic linear relationship. All comparison results should be considered in the context of limitations of small cohort size.

## Data Availability

• The published article includes all datasets generated or analyzed during this study. • This paper does not report original code. • Any additional information required to reanalyze the data reported in this paper is available from the lead contact upon request.
