# Supplementary material for: Serum concentration of antigen-specific IgG can substantially bias interpretation of antibody-dependent phagocytosis assay readout
Source: iScience. 2023 Aug 3;26(9):107527. doi: 10.1016/j.isci.2023.107527 (PMC10469534; doi:10.1016/j.isci.2023.107527)
Supplement: Supplementary file 1 — Document S1. Figures S1 and S2 [file mmc1.pdf]

iScience, Volume *26*

Supplemental information

Serum concentration of antigen-specific IgG can substantially bias interpretation of antibody-dependent phagocytosis assay readout

Russell St. Germain, Emily L. Bossard, Lawrence Corey, and Anton M. Sholukh

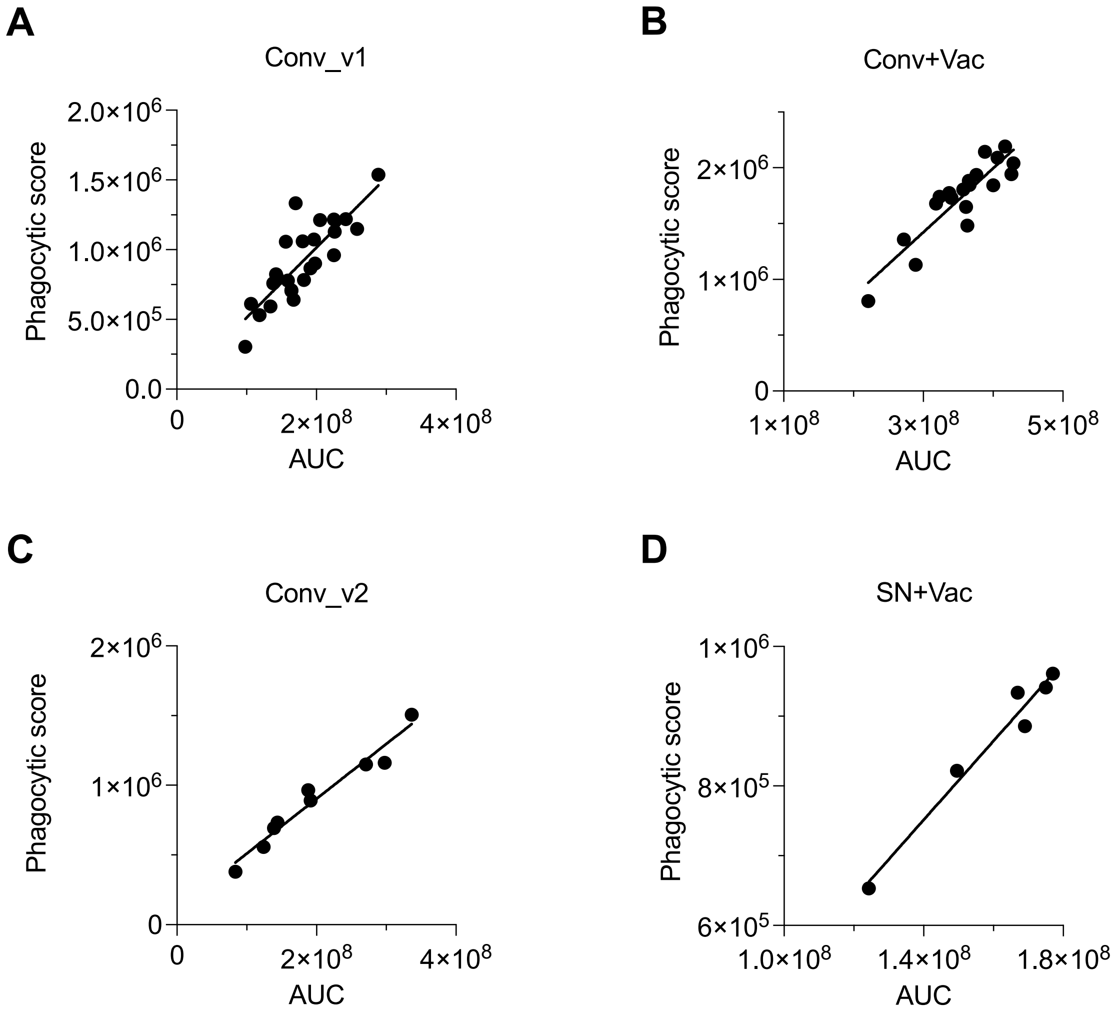

Supplemental Figure 1. **Deming linear regression and Pearson correlation for AUC and phagocytosis score at 50 ng/ml of spike-specific IgG, Related to Figure 3.** (**A**), Samples collected at 2-months post-COVID-19 (n=25). (**B**) Samples collected after one dose of mRNA vaccine in previously infected participants (n=19). (**C**) Samples collected after 2 doses of mRNA vaccine in seronegative subjects (n=6). (**D**) Samples collected at 9-months post-COVIDS (n=9). Values for Pearson's r and two-tailed *P* are depicted on graph inserts.

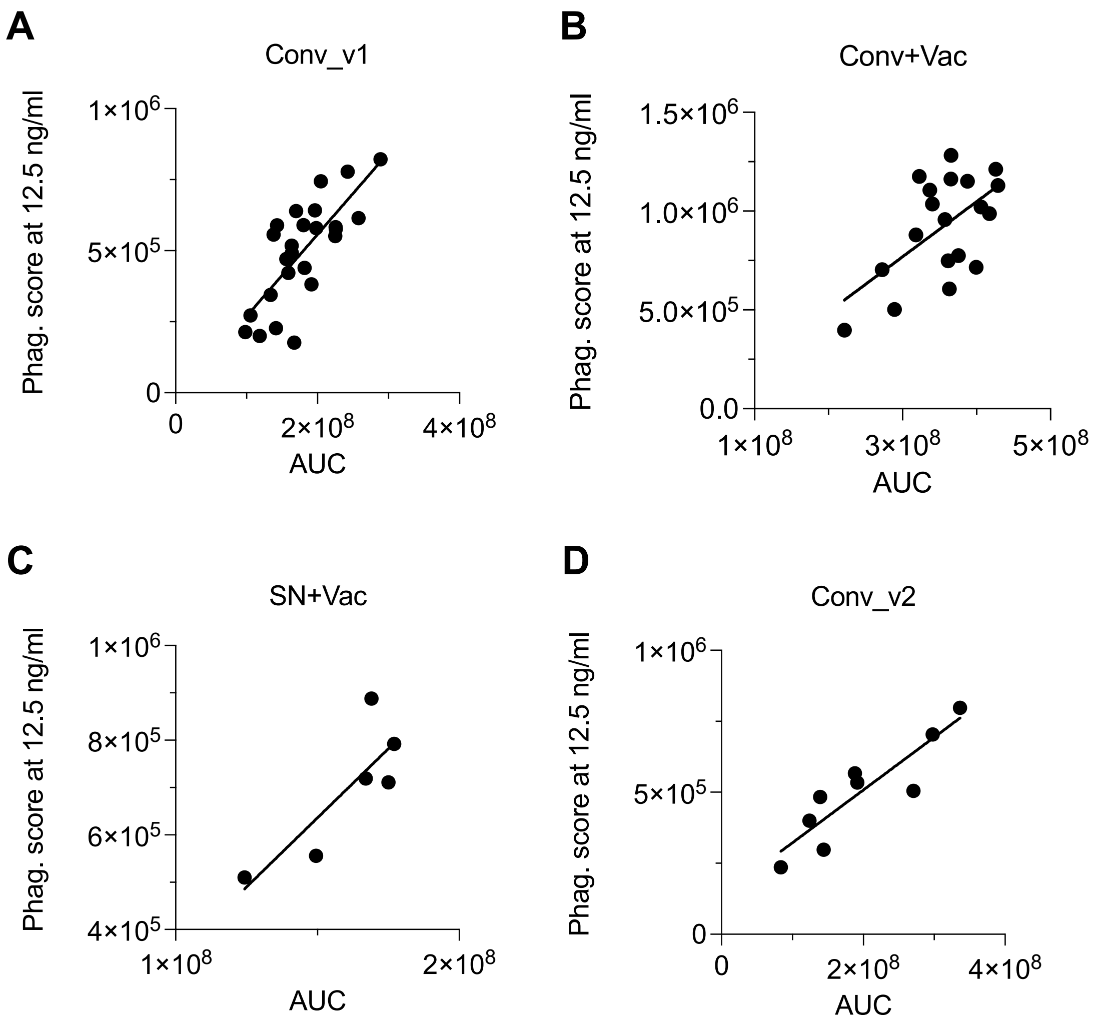

Supplemental Figure 2. **Deming linear regression and Pearson correlation for AUC and phagocytosis score at 12.5 ng/ml of spike-specific IgG, Related to Figure 3.** (**A**) Samples collected at 2-months post-COVID-19 (n=25). (**B**) Samples collected after one dose of mRNA vaccine in previously infected participants (n=19). (**C**) Samples collected at 9-months post-COVIDS (n=9). (**D**) Samples collected after 2 doses of mRNA vaccine in seronegative subjects (n=6). Values for Pearson's r and two-tailed *P* are depicted on graph inserts.
